# Hospital Notes

**Published:** 1902-02

**Authors:** 


### HOSPITAL NOTES.

THE Buffalo Hospital of the Sisters' of Charity medical and surgical staff has elected the following-named physicians as officers or the year 1902: President, H. C. Buswell; vice-president, Walter D. Greene; secretary, Max Keiser; executive committee, E. J. Meyer, James J. Mooney and Charles S. Jewett.

THE New Emergency Hospital was recently opened for the reception of patients. The following-named physicians constitute the staff: Medicine, John H. Pryor, L. Bradley Dorr; Surgery, C. M. Daniels, F. J. Carr, Vertner Kenerson; ophthalmology, H. Y. Grant, B. H. Grove; laryngology, rhinology and otology, W. Scott Renner, E. A. Forsyth; dermatology, Ernest Wende; genito-urinary, James A. Gardner, N. W. Wilson; nervous diseases, William C. Krauss; consulting chemist, John A. Miller, M.S., Ph.D., F.C.S. (University of Berlin); internes, C. W. Southworth, J. H. Dewees, G. McKay Hall.

THE German Hospital staff held its annual meeting Thursday, January 9, 1902, at which the following officers of the staff were reëlected: President, C. H. W. Auel; vice-president, M. Hartwig; secretary, C. Weil.

